# The ST2/Interleukin-33 Axis in Hematologic Malignancies: The IL-33 Paradox

**DOI:** 10.3390/ijms20205226

**Published:** 2019-10-22

**Authors:** Alessandro Allegra, Vanessa Innao, Gennaro Tartarisco, Giovanni Pioggia, Marco Casciaro, Caterina Musolino, Sebastiano Gangemi

**Affiliations:** 1 Division of Hematology, Department of Human Pathology in Adulthood and Childhood, University of Messina, 98125 Messina, Italy; vinnao@unime.it (V.I.); cmusolino@unime.it (C.M.); 2 National Research Council of Italy (CNR)-Institute for Biomedical Research and Innovation (IRIB), 98164 Messina, Italy; gennaro.tartarisco@cnr.it (G.T.); giovanni.pioggia@cnr.it (G.P.); 3 School and Division of Allergy and Clinical Immunology, Department of Clinical and Experimental Medicine, University Hospital “G. Martino”, Via Consolare Valeria SNC, 98125 Messina, Italy; mcasciaro@unime.it (M.C.); gangemis@unime.it (S.G.)

**Keywords:** alarmin, hematologic malignancies, interleukin 33, immune response, tumorigenesis

## Abstract

Interleukin (IL)-33 is a chromatin-related nuclear interleukin that is a component of IL-1 family. IL-33 production augments the course of inflammation after cell damage or death. It is discharged into the extracellular space. IL-33 is regarded as an “alarmin” able to stimulate several effectors of the immune system, regulating numerous immune responses comprising cancer immune reactions. IL-33 has been demonstrated to influence tumorigenesis. However, as far as this cytokine is concerned, we are faced with what has sometimes been defined as the IL-33 paradox. Several studies have demonstrated a relevant role of IL-33 to numerous malignancies, where it may have pro- and—less frequently—antitumorigenic actions. In the field of hematological malignancies, the role of IL-33 seems even more complex. Although we can affirm the existence of a negative role of IL-33 in Chronic myelogenos leukemia (CML) and in lymphoproliferative diseases and a positive role in pathologies such as Acute myeloid leukemia (AML), the action of IL-33 seems to be multiple and sometimes contradictory within the same pathology. In the future, we will have to learn to govern the negative aspects of activating the IL-33/ST2 axis and exploit the positive ones.

## 1. Introduction

### 1.1. IL-33

Interleukin (IL)-33 is a component of the IL-1 family, cytokines that maintain a structure of β-trefoil folds containing 12 antiparallel β-strands that are organized in a three-fold symmetric configuration [1]. IL-33 was originally reported as a protein amply present in endothelial venules that facilitates the entrance of lymphocytes into lymphoid organs, and was hence called “nuclear factor from high endothelial venules” [2].

It is now recognized that this cytokine is a chromatin-related nuclear interleukin, and that connecting to histones is crucial for IL-33 action [3]. Nuclear IL-33 can act as a transcriptional repressor when overexpressed in cells [4]. IL-33 production can be augmented in the course of inflammation [2,5]. In fact, after cell damage or death, IL-33 is discharged into the extracellular space and operates as an endogenous danger signal that warns the immune cells of the occurred damage. The liberation of IL-33 can be due to necrotic cell death [5].

**Figure 1:**
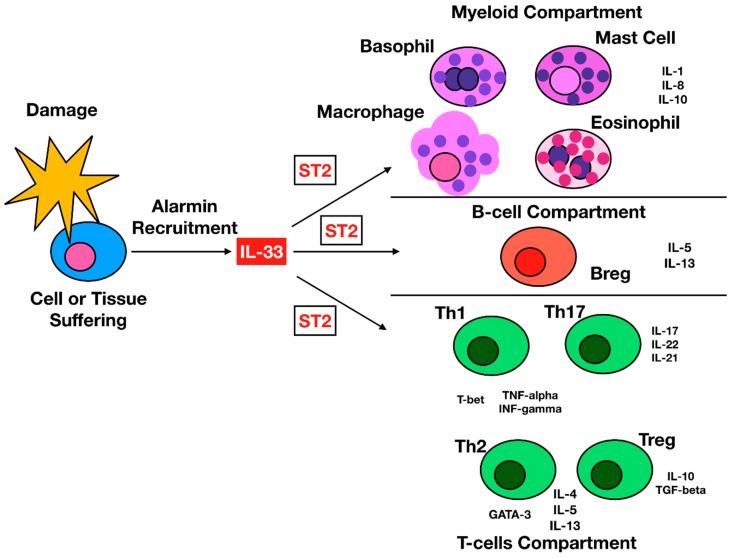
IL-33 Interleukin-33 (IL-33) signaling on immune cells. Tissue and cells damage lead to the release of IL-33 from these cells. IL-33 then signals to many different immune cells, enhancing their function and activating several immune mediators involved in inflammation and immune regulation.

IL-33 is regarded as an “alarmin” able to stimulate several effectors of the immune system, regulating numerous immune responses comprising cancer immune reactions [6,7] (Figure 1).

The precise processes which lead to the release of IL-33 are yet not completely known, and it may be different in diverse experimental models. In fact, in addition to the classical damage liberation of IL-33, the cytokine may be released after stimulation of purinergic receptor P2Y2R signaling [8]. Furthermore, alternative splicing-mediated deletion of exon 3 and 4 of IL33 transcripts provoke cytoplasmic delocalization of IL-33 proteins, and this isoform can maintain its capacity to react with its receptor [9].

IL-33 is produced in a full-length configuration (270 amino acids), and it gets cleaved by neutrophil serine proteases cathepsin G and elastase once it is released from the nucleus, which increases its activity 10-fold [10]. However, it was largely demonstrated that this activation process is not necessary for IL-33 to exert its function because it has different bioactive forms [2,3,11]. It is of interest that during apoptosis, a type of cell death that does not activate inflammation, IL-33 is deactivated by endogenous caspases [11,12,13]. A different system able to control IL-33 action is oxidation. Extracellular IL-33 is vulnerable to cysteine oxidation that causes the formation of disulphide bridges, determining conformational modifications that impede the link to its receptor, thus inactivating IL-33 [14].

However, the presence of diverse human full-length mRNA splice variants has been demonstrated, according to the cell type and the disease, and different immune effects are caused by the different molecules [15,16]. Moreover, inflammatory proteases from mast cells and other cells can transform full-length IL-33 into shorter structures (18–21 kDa) whose action is 30-fold more potent than the full-length form. However, the shorter form does not pass into the nucleus as it does not have the nuclear localization signal present in full-length IL-33 [10,17,18,19].

### 1.2. The ST2 Receptors

**Figure 2:**
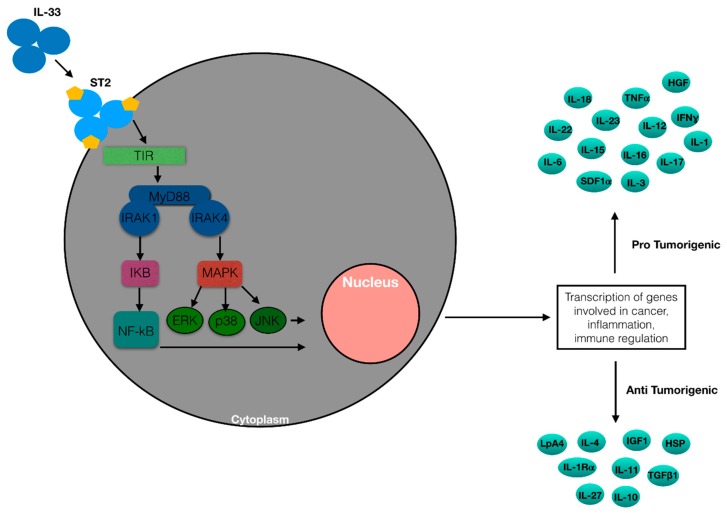
ST2/IL-33 immune cells signaling pathway. IL-33 either binds to the ST2 receptor, recruiting MyD88 to its intracellular domain. MyD88 binding recruits IL-1R-associated kinase (IRAK) 1 and 4, leading to either the NF-κB or MAPK pathway being activated. Their activations promote inflammatory cytokine expressions. ST2/IL-33 signaling has been shown to promote Treg and immune system cells function and expansion through enhancing TGF-β1-mediated differentiation though a p38-dependent mechanism. ST2/IL-33 link activates the transcription of several genes involved in tumorigenesis, inflammation and immune regulation.

IL-33 is a ligand of its receptor, which is composed of ST2/IL1RL1 and IL1RAP. It is encoded by Interleukin 1 receptor-like 1 (IL1RL1) that generates four different isoforms via alternative splicing: ST2 ligand (ST2L), sST2, ST2 variant (ST2V), and ST2 ligand variant (ST2LV). ST2L is a membrane receptor that contains an ILI-R1-like intracellular domain, a transmembrane spanning region, and three Ig-likeextracellular domains [20,21]. sST2, a glycosylated protein, is a soluble form of ST2. It does not have the transmembrane domain but includes an extracellular domain analogous to ST2L and nine amino acids at the C-terminal tail [22]. ST2V is similar to sST2 but includes a hydrophobic tail instead of the third Ig-like domain [23,24]. ST2LV is an N-glycosylated and soluble form that does not have the transmembrane domain present in ST2L [25]. ST2L creates a heterodimeric transmembrane receptor complex with the IL1-receptor accessory protein, IL1-RAcP, which is required for signaling after IL-33 binding [26], and sST2 works sequestering extracellular IL-33 (Figure 2).

Expression of the IL1RL1 gene is controlled by GATA1/2 and estrogen-response elements (EREs) that are found in the distal and proximal promoters that rule ST2L and sST2 expression [27,28,29,30,31].

### 1.3. IL-33 Cellular Targets

Most hematopoietic cells present the ST2 receptor: T helper (Th)1 cells, Th2 cells, innate lymphoid cells 2 (ILC2s), CD8+ T cells, some Treg cells, natural killer (NK) cells, mast cells, B cells, neutrophils, basophils, eosinophils, and myeloid-derived suppressor cells (MDSC) are cells that express high levels of ST2. However, nonhematopoietic cells, comprising fibroblasts, epithelial cells, endothelial cells, and dendritic cells (DC), also express ST2 and may respond to IL-33 [32,33,34].

Regarding the effects of IL-33 on the main cellular effectors, stimulation with IL-33 pushes naïve CD4+ T helper cells differentiation toward a Th phenotype [35]. Human CD4+ T cells can have antitumor activity by expressing high amounts of MHC class II and self-antigens on their membrane via secretion of IFN-γ [36]. Mousa Komai-Koma et al. demonstrated that IL-33 may increase CD4^+^ Th1 differentiation via a mechanism depending on IL-12 and ST2. IL-33 and IL-12 synergistically augment ST2 expression in early activated CD4^+^ T cells, while it is ineffectual on mature Th1 cells [37]. This is because ST2 expression is produced only in early-TCR activated naïve CD4^+^ T cells and is proresively inhibited in mature Th1.

ST2/IL-33 signaling is also able to increase suppressive CD4^+^ Foxp3^+^ GATA3^+^ Treg cells in vivo and in vitro [38]. IL-33-expanded Tregs produce strong suppressor action in several diseases [39].

ST2^+^ Treg increase can be due to IL-33 signaling in DCs, via the generation of IL-2 [40]. In the intestine, IL-33 signaling increases transforming growth factor (TGF)-β1-mediated transformation of Treg cells and acts as a signal for Treg accumulation in damaged tissues [41], while in Apc^Min/+^ mice, epithelial-derived IL-33 increase the proliferation of ST2^+^ Treg cells [42,43,44].

Regarding CD8^+^ T cells, only effector or polarized type 1 cytotoxic T (Tc1) cells express ST2, while naïve and early activated CD8^+^ T cells lack the receptor [45]. Expression of ST2 in CD8^+^ T cells is controlled by T-bet, a master transcription regulator, and it has been reported that IL-33 augments T-bet and Blimp1, transcription factors crucial for CD8^+^ T cell [45].

IL-33 stimulates NKT and NK cells, provoking IFN-γ production through IL-12 signaling [46]. In vitro, IL-33 increases NK cell cytotoxicity, and upregulated CD69 expression [46]. In animal models, transgenic expression of IL-33 augmented the accumulation of cytotoxic NK cells to the tumor tissue that inhibited metastasis formation [46]. Furthermore, augmented rates of CD107a^+^IFN-γ^+^ NK cells were described after IL-33 administration in spleens of B16 melanoma-bearing mice [47]. In contrast with these studies, investigations performed in breast cancer model demonstrated that IL-33/ST2 alters NK cell activation. ST2-deficient animals bearing breast tumors displayed augmented activated NK cells and NK cytotoxic activity and enhanced antitumor activity in ST2^−/−^ mice [48]. Moreover, exogenous administration of IL-33 to wild type breast tumor-bearing animals reduced NK cytotoxicity and increased cancer progression [49].

Mast cells (MC) are innate immune cells. MCs present the IL-33 receptor, and IL-33 stimulates MCs. However, whether IL-33 plays a role in MC survival remains to be proved. However, in skin-derived human MCs, IL-33 decreased MC apoptosis without modifying growth via the action of the antiapoptotic molecule B-cell lymphoma-X large [50].

**Figure 3:**
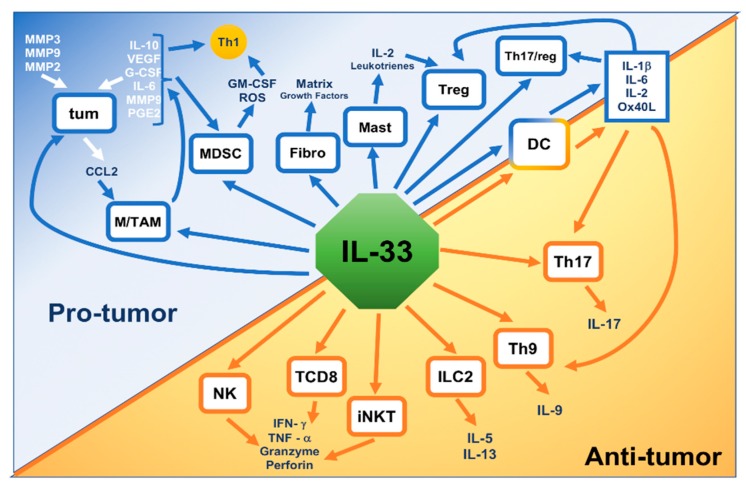
Dual role of IL-33 in cancer.IL-33 released in the TME is able to stimulate cancer cells (tum), fibroblasts (Fibro), and different immune cells (Macrophages, TAM, MDSC, mast cells, Treg, Dendritic Cells, Th 17, Th 9, Group 2 Innate lymphoid cells (ILC2), invariant Natural Killer cells (iNKT), CD8+ T cells, and Natural Killer cells) which are activated to produce molecules involved in protumor or antitumor processes leading to the development or to the regression of the tumor. Some cytokines produced by protumor cells such as myeloid-derived suppressor cells (MDSC) or Tumor-associated macrophages (TAM), are also able to produce cytokines which inhibit antitumor cells such as all the Th1 cells.

Myeloid-derived suppressor cells (MDSCs) are lacking in normal subjects but increase in neoplastic diseases, where they have an immune-suppressive action [51]. Numerous works reported the capacity of IL-33 to increase MDSCs during the onset of tumors. In animal tumor models, IL-33 has been described to increase MDSC growth. IL-33 augmented the intratumoral increase of MDSCs that presented TGF-β1 and IL-13α1R, while the absence of IL-33/ST2 signaling decreased the growth and the immunosuppressive capacity of MDSCs. Moreover, IL-33 increased activity of arginase-1 in MDSCs and caused activation of MAPK and NF-κB, with an increase of their immunosuppressive effects [52,53]. The action of IL-33 on all the effector cells reported above is partially due to its effecton cytokine production. It was demonstrated that IL-33 provokes production of several cytokines, including IL-4, IL-5, IL-6, IL-8, IL-10, IL-13, MIP-1α, IP-10, MCP-1, TNF, and GM-CSF [17,54,55] (Figure 3).

### 1.4. IL-33 Action in Nonhematologic Malignancies

The cancer microenvironment is a crucial factor for tumor onset. This milieu can be modified by several elements comprising pro- or antitumorigenic immune cells and cytokines [56].

Inflammation may have a protumorigenic activity. About 15% of cancers are linked to a conditions of chronic inflammation [57].

The alarmin IL-33 has been demonstrated to participate in several forms of inflammatory diseases and to influence tumorigenesis. Firstly, IL-33 was intended to be a procancer mediator, since the activation pathway of IL-33/ST2 could promote metastasis. After this first period, it emerged how by the activation of the immune system it could stimulate immune surveillance, thus exerting an antitumorigenic function [58,59].

However, as far as this cytokine is concerned, we are faced with what has sometimes been defined as the IL-33 paradox. Several data have demonstrated a relevant role of IL-33 in numerous malignancies, where it may have both pro- and—less frequently—antitumorigenic actions [60,61].

Dependent on the cancer milieu, IL-33 may influence anticancer immunity, stimulating CD8-positive T cells essential for the elimination of tumor cells. Th17 and Treg cells are also immune modulators in cancer, and Th17 cells influence antitumor responses, while Treg cells have an action in the conservancy of self-tolerance and in the regulation of immune response to cancer cells [61]. Protumor action can also be exerted by the effect of the cytokine on MDSCs.

Some data have reported a positive association between IL-33 production in cancer cells and the best prognosis in tumor subjects, where IL-33 and ST2 concentrations were downregulated in cancer lung cells [62]. Moreover, plasma IL-33 concentrations were increased in the course of the early stage of lung carcinoma and reduced in the advanced stage of disease [63,64]. IL-33 levels were demonstrated to be a positive prognostic marker in hepatocellular carcinoma and in breast cancer cells [65,66]. IL-33, together with its receptor ST2, seems to play a key role in the tumor environment by involving different pathways, such as interferon regulatory factor-3 (IRF-3), MyD88, AKT, COT, ERK, and JNK.IL-33 may act in both a pro- and antioncogenic way by increasing cells’ metabolism and by regulating the immune system [61,67] (Table 1).

## 2. IL-33 and Hematologic Malignancies

### 2.1. BCR-ABL1-Negative and Positive Myeloproliferative Neoplasms.

Myeloproliferative neoplasms (MPNs) comprise a group of malignant hematopoietic conditions that consist of BCR-ABL1-negative diseases such as polycythemia Vera (PV), essential thrombocythemia (ET), and primary myelofibrosis (PMF), as well as BCR-ABL1-positive diseases such as chronic myelogenous leukemia (CML).

In an animal model of MPN, IL-33 has been demonstrated to have a crucial action in stimulating altered myelopoiesis. Animals that are homozygous for a mutant allele of inositol polyphosphate-5-phosphatase D (Inpp5d), named styx, repeat the genesis of the MPN-like disease in Inpp5d knockout mice. Deficiency of MyD88 and Irak4 annulled disease progress. MyD88 and Irak4 transduce signals through Il-33, IL-1, and IL-18. Nevertheless, only the deletion of IL-33 was sufficient to re-establish normal hematopoiesis [68], while IL-33 produced from stromal cells increased the production of growth factors in bone marrow to cause myeloproliferation in mice. Augmented rates of IL-33-expressing cells were reported in bone samples from MPN subjects, and IL-33 administration stimulated colony formation by CD34+ MPN progenitor cells from patients [68]. Thus, IL-33 seems to be a crucial element in the onset and progression of MPN, although the interpretation of existing data is not easy to read. For instance, the role of IL-33 seems to be relevant for other specific chronic Philadelphia negative myeloproliferative diseases, but with a peculiar pattern. PV and ET patients had decreased plasma IL-33 levels that were thought to alter their immune system [69]. Probably, these difference could be explained by the hypersensitivity of MPN stem/progenitor cells to cytokines and growth factors, and it is plausible that even low amounts of secreted IL-33 within the niche may be sufficient to start a ST2-dependent inflammation that favors MPNs.

Regarding BCR-ABL1-positive myeloproliferative neoplasms, CD34+ cells from CML patients were targeted by IL-33. After IL-33 administration, these cells displayed an augmented expression of ST2, and an increased proliferation, while progenitor cells from control subjects did not and were insensitive to IL-33. Alteration of the IL-33/ST2 pathway was a result of the tyrosine kinase activity by the BCR-ABL1 oncogene. In experimental models, engraftment of CD34+ bone marrow progenitor cells from humans and animals expressing BCR-ABL1 was less effective in IL-33 knockout mice versus wild type animals, implying that IL-33 is essential for CD34+ progenitor proliferation [68,77].

ST2 overexpression is regularized after imatinib mesylate treatment, while IL-33 neutralizes in vitro imatinib mesylate-induced proliferation arrest in CML CD34 progenitors through activation of the STAT5 pathway. Concentrations of circulating soluble ST2, generally believed to be a functional sign of IL-33 signaling in vivo, correlate with disease load. The increased levels associated with a high Sokal score predictive of prognosis are normalized in CML subjects in molecular remission. Lastly, an effect of IL-33 on maintenance of CD34+ cells from CML subjects was demonstrated by using xenotransplant experiments in immunodeficient NOD Shi-SCID IL2Rγc^nul^ (NOG) mice [77]. These data demonstrated that CML cells were sensitive to IL-33, which was capable of sustaining proliferation and cytokine production both directly, indirectly by the expression of ST2, and by controlling the neoplastic microenvironment, suggesting a new therapeutic approach.

Tare et al. identified a basophil-like chronic myelogenous leukemia cell line, KU812, that presents ST2L and has a response to IL-33 stimulation. IL-33 caused generation of several inflammatory mediators that were reduced by anti-ST2L and anti-IL-33 antibodies [78].

We must also consider that the IL-33/ST2 system in the BM can be stimulated both in hematopoietic cells and in stromal/nonhematopoietic cells. Activation of ST2 on these cells provokes the production of several cytokines (IL-6, GM-CSF, G-CSF, and IL-3) that can activate the STAT5 pathway and can cause resistance to imatinib mesylate [68,82].

Finally, the possible role played by IL-33 in the onset of side effects of tyrosine kinase inhibitors also appears to be of some interest. As reported in a previous case report belonging to our group, imatinib mesylate-related symptoms of dermatologic toxicities might be determined by the discharge of IL-33. In particular, it is possible that TKi treatment could provoke keratinocyte damage, the release of IL-33, and the successive contact of IL-33 with ST2 receptor on mast cells that causes the liberation of numerous factors able of causing skin lesions, comprising IL-31, a known pruritus-inducing cytokine [83].

While these results suggest a possible therapeutic advantage in stopping IL-33/ST2 signaling in MPNs, other studies should determine the role of this system for disease progression and drug resistance in MPN subjects. Another option could comprise a multitarget approach, involving IL-33 and ST2 blockade together with some other cytokines involved in the resistance pathway (IL-6, GM-CSF, G-CSF, and IL-3). It may be relevant in the future to address whether some in vitro experimental blockade could give some therapeutic advantages.

### 2.2. Acute Myeloid Leukemia

Although our comprehension of the possible immunotherapy approaches to treat acute myeloid leukemia (AML) is still growing, it is evident that leukemia cells have developed several diverse immunosuppressive activities to elude the antileukemia responses (increase of Tregs and MDSC, upregulation of programmed death 1 (PD-1) [84]. However, recent data demonstrated the main mechanism of the immune escape is the tolerance of leukemia-specific CD8+ T cells [85,86,87,88], and IL-33-mediated actions on leukemic cells could be due to CD8+ T cells and MDSC populations as speculated and demonstrated in other tumor experimental models [52,74].

Employment of recombinant IL-33 markedly reduces murine AML cell proliferation and increases survival. Notably, IL-33 administration licensed DCs from tumor-bearing mice to overwhelm the tolerance of leukemia-specific CD8+ T cells by causing expression of several costimulatory molecules. Moreover, IL-33 considerably augmented the effectiveness of PD-1 blockade, causing complete regression of disease in almost half of the treated animals. This suggests the possibility of a novel therapeutic approach of leukemia immunotherapy [75].

Studies have evaluated the existence of possible correlations between genetic alterations and the functioning of the ST2/IL33 system. In AML patients, chromosomal abnormalities have been reported. One of the most frequent alterations, inversion of chromosome 16 [inv (16)], produces the fusion oncogene CBFB-MYH11. This oncogene induces expression of IL1RL1. Employing Cbfb-MYH11 knock-in mice, it was reported that ST2 is present on cells with high leukemia stem cell activity. Moreover, ST2^+^ cells can survive chemotherapy better than ST2^−^ cells in vivo [82]. Levescot et al. made a similar observation for BCR-ABL1 in CML [77]. The clinical significance of such data remains to be demonstrated.

IL-33 also stimulates basophilic differentiation in normal cells but appears to be more effective in patients with hematopoietic malignancies. Generally, basophil differentiation does not appear to depend on a specific growth factor or cytokine. Although IL-33 is a powerful driver of basophil differentiation in vitro, IL-33 knockout animals do not display an alteration in these cells. The expression of GATA2 is essential for appropriate basophil differentiation, but they are supposed to operate with other transcription elements, including GATA1 and STAT5 [9,13,89]. Acute basophilic leukemia (ABL) is an infrequent form of AML. In previous works, a recurrent t (X; 6) (p11; q23) translocation generating an MYB–GATA1 fusion gene was described in ABL patients. Cells presenting the chimeric MYB–GATA1 transcription factor displayed augmented expression of CD34 (a marker of immaturity) and of Fc RI and CD203c (markers of basophilic differentiation). IL-33 augmented the basophilic differentiation of MYB–GATA1-expressing cells, confirming the relevance of the ST2/IL-33 system in the basophilic differentiation of leukemic cells and CD34-positive primary cells [76]. Results showed that IL-33 effects in normal cells are rather modest and this suggests that this cytokine could be more effective in malignant cells than in normal ones, confirming once more the fundamental role of the IL-33/ST2 axis in hematologic malignancies.

### 2.3. Lymphoproliferative Diseases

The relevance of IL-33 in lymphoproliferative diseases has been demonstrated in animal models and studies conducted on human patients.

Genetic aberrations in Notch1 and Notch2 arise in B-cell neoplastic diseases such as Hodgkin lymphoma (HL), diffuse large B-cell lymphoma (DLBCL), chronic lymphocytic leukemia (CLL), follicular lymphoma (FL), and mantle cell lymphoma (MCL). B cells presenting altered Notch1 signaling have an immunomodulatory action on T cells by intensifying Th2 and Treg response in an IL-33-dependent manner. An experimental animal model, in which expression of Notch1 is provoked in B cells by AICDA gene promoter-driven CRE recombinase, demonstrated an increase of Th2 and Treg cells and a diminished production of cytokines by Th1 and CD8 T cells. Animals were more susceptible to tumors, indicating an altered antitumor T-cell capacity. This modified T-cell response was due to augmented IL-33 production by Notch1-activated B cells. Animals knockout of IL33 or the use of antibodies to block Il-33 annulled the Treg and Th2 cell response stimulated by B cells. Gene expression results obtained from patients affected by diffuse large B-cell lymphoma demonstrated that activated Notch signaling correlates positively with IL33 expression [70].

However, different experimental data seem to come from studies performed on Burkitt’s lymphoma (Daudi cells) as demonstrated by the effect of IL-33 on γδ T lymphocytes. γδ T cells presenting the Vγ9 T cell receptor are the more represented γδ T-cells in peripheral blood. After stimulation with phosphoantigens (PAg), these cells multiply and discharge proinflammatory cytokines, and drive cell cytotoxicity against several neoplastic diseases [71]. An increased production of PAgs by Burkitt’s lymphomas mean that the neoplastic cells are identified and destroyed by the Vγ9 T cells [90,91]. IL-33 was able to provoke the in vitro growth of PAg-stimulated Vγ9 T cells, which were displaying in vitro antitumor cytotoxicity. Human Vγ9 T cells were expanded in vivo (in NSG mice) by IL-33. Hence, IL-33 could represent a substitute of IL-2 for Vγ9 T cell-based cancer immune treatments [72].

Regardinghuman patients with lymphoproliferative diseases, we evaluated the plasma concentrations of IL-33 in 77 subjects with B-CLL. There was a relevant variation between the CLL levels of IL-33 versus controls, with a reduction of IL-33 concentrations in patients. Moreover, there was a difference, although not statistically relevant, between the IL-33 concentrations in CLL subjects before and after treatment. Finally, a negative correlation between IL-33 and CD20 expression and a positive correlation between IL-33 and CD3 expression were also reported [92].

In LLC subjects, IL-33 seems to drive Th2 responses. Podhorecka et al. studied the Th1/Th2 balance in CLL subjects and proved the dominance of T type 1 cells and T cell-mediated immunity that is changed towards type 2 during the phase of disease progression [93]. The decrease in plasma concentrations of IL-33 might therefore justify the altered Th2 response described in these subjects [73]. Our study described the existence of an inverse correlation between plasma levels of IL-33 and CD20 expression, and IL-33 seems to be able to modify the expression of CD20. The exact mechanism leading to this process remains to be clarified [92].

Finally, the datum that the treatment of CLL provokes a normalization of IL-33 plasma concentrations suggests a direct role of the disease on the level of this cytokine. The contrasting data about IL-33 levels in patient serum could be both correlated to the rather short half-life of the molecule or to the fact that in some kinds of tumors it is secreted nearby its niche of near the neoplastic mass (i.e., lung cancer). Moreover, it should be considered the dual role of the alarmin, which on one side stimulates immune tolerance, on the other activates the proinflammatory cascade, giving rise to some oncogenic pathways.

### 2.4. Monoclonal Gammopathies

There are numerous possible pathogenetic and therapeutic implications related to the role of IL-33 in patients with monoclonal gammopathies. For instance, our team investigated the plasma concentrations of IL-33 in 44 multiple myeloma (MM) subjects and 13 subjects with monoclonal gammopathy of undetermined significance (MGUS). IL-33 concentrations were statistically decreased in MM and MGUS subjects versus healthy controls. In MM subjects, a negative correlation between IL-33 level and stage was found [94].

Decreased IL-33 concentrations might justify the altered T response described in MM. Moreover, these data may imply that during disease evolution or the shift from a benign condition (MGUS) to a neoplastic condition (MM), the deficiency in cytokine production aggravates gradually, causing a further alteration of the immune responses [95].

IL-33 could have a role in bone disease of MM subjects. Different experiments demonstrated that in vitro IL-33 reduced osteoclast precursors’ differentiation into mature osteoclasts and decreased their bone resorptive action. This datum is consistent with the hypothesis that IL-33 inhibits osteoclastogenesis [79,80], and is confirmed by other studies. For instance, it was described that curdlan, an agonist of dectin-1, reduces osteoclastogenesis. Zhu et al. reported that curdlan strongly reduced RANKL-induced osteoclast differentiation and bone resorption. Curdlan impeded the expression of nuclear factor of activated T-cells, cytoplasmic 1 (NFATc1), a crucial transcriptional factor for osteoclastogenesis. Remarkably, dectin-1 activation augmented the expression of Musculoaponeurotic Fibrosarcoma oncogene omolog B (MafB), an inhibitor of NFATc1, and IL-33 in osteoclast precursors. The same study demonstrated that IL-33 augmented the expression of MafB in osteoclast precursors and reduced the differentiation of osteoclast precursors into mature osteoclasts. Moreover, stopping ST2 partially abolished curdlan-induced inhibition of NFATc1 expression and osteoclast differentiation [81]. Once more, the paradoxical role of the IL-33 emerges, by enlightening its ability to block cell proliferation in particular situations in experimental models. However, blocking the IL-33/ST2 pathways in vitro could show its limits due to the different biological activity of the diverse in vivo IL-33 variants [96].

## 3. Conclusions

### 3.1. Future Challenges and Perspectives

IL-33 is a cytokine involved in the regulation of not only antitumor immunity but tumor growth. Immune responses can be activated by tumor growth via emitting danger signals [97]. Nevertheless, tumors progress by modifying the action of immune suppressive cells such as Treg and MDSc [98,99,100].

**Figure 4:**
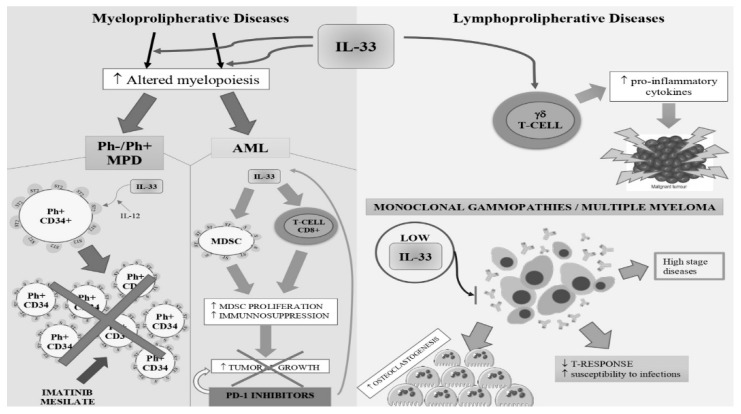
A summarizing scenario of IL-33’s role in hematological malignancies. IL-33’s pro- and antitumorigenic role is reported as follow. In MPN, IL-33 was demonstrated to have a crucial action in stimulating altered myelopoiesis. IL-33 increases the production of growth factors in bone marrow and causes myeloproliferation. As a consequence, there are augmented rates of IL-33+ in MPN subjects, and IL-33 administration stimulates colony formation from CD34+ MPN progenitor cells. In acute myeloid leukemia (AML), cells develop several diverse immunosuppressive activities to elude the antileukemia responses (increase of Tregs and MDSC, upregulation of programmed death 1 (PD-1). On the contrary, in lymphoproliferative diseases, augmented levels of the alarmin could favorite a proinflammatory response against the tumor. In multiple myeloma (MM), low levels of IL-33 are correlated with higher disease stages, minor T-response, and augmented osteoclastogenesis.

As noted in the previous paragraphs the pro- or antitumor role of cytokines varies according to the neoplastic pathology considered. In the field of hematological malignancies, the role of IL-33 seems even more complex. Although we can affirm the existence of a negative role of IL-33 in CML and in lymphoproliferative diseases and positive in pathologies like AML, the action of IL-33 seems to be multiple and sometimes contradictory within the same pathology. For example, in the course of MM, the reduction of IL-33 could harm the susceptibility to infections and a positive action on bone disease. However, we tried to give a clearer scenario by summarizing IL-33’s potential role in hematological malignancies (Figure 4). In the future, we will have to learn to govern the negative aspects of activating the IL-33/ST2 axis and exploit the positive ones.

Upcoming studies on the effects of IL-33 on malignancies will probably focus on promotion of NK cell infiltration, group 2 innate lymphoid cells proliferation, and DC activation [101].

An intriguing topic will also be the nuclear function of IL-33. In fact, in addition to its extracellular role as an alarmin activating ST2 signaling, IL-33 also has a nuclear function [4]. This nuclear action of IL-33 may justify why some studies performed employing St2-deficient mice may not repeat the results from IL33^−/−^ animals. Furthermore, the study of the nuclear IL-33 action will be useful to evaluate the possible interactions with tumor-related transcription factors, and the modulation of nuclear IL-33 expression could represent a possible therapeutic approach.

For its crucial role in tissue damage-induced tumorigenesis, IL-33 is a potential target, and diverse methods could be proposed to use or to target the cytokine. The employment of IL-33 as an adjuvant has been contemplated to regulate Th2 polarization [81]. Even in this case, however, prudence appears necessary due to the many target cells and the possibility to increase tumor progression in chronic myeloid malignancies. Neutralizing antibodies targeting IL-33 or ST2L could be utilized to reduce the activity of IL-33. However, in this area, their utilization may be checked due to the oxidation of cysteine residues and the existence of short-acting IL-33 within tissue microenvironments, with an underestimation of IL-33 concentrations due to an incapacity to evaluate IL-33 before degradation [102].

Moreover, therapeutic targeting of IL-33 may enhance the efficacy of the therapy with conventional treatment. For example, a sequence of imatinib and IL-33 blockade in CML may lead to eradication of cytokine-dependent malignant stem cells and avoid the appearance of drug resistance. In an experimental mouse model, the combination of IL-33 and PD-1 blockade was reported to enhance the survival of animals suffering from AML [75,103].

A different field for a novel employment of IL-33 could be the vaccine therapy of neoplasms [104,105], as IL-33 works as a promoter of memory T cell immunity [106,107].

Finally, in the coming years, new methods of cytokine intervention will be developed. Ramadan et al. described several small-molecule ST2 inhibitors identified via a combination of high-throughput screening and computational analysis. After in vitro and in vivo toxicity evaluation, three molecules were chosen for validation in experimental GVHD models. The most effective molecule, iST2-1, decreases plasma sST2 concentrations, enhances survival, and preserves graft-versus-leukemia action [108]. Augmented circulating sST2 has been linked to pathological states. Evaluating this soluble molecule could be as important as dosing IL-33 in order to follow disease progression. Hematological malignancies are not an exception to this statement. Further studies on diverse and larger cohorts of patients should be considered in order to confirm their prognostic value.

The introduction of the modulation of IL-33 activity into clinical practice is certainly still a premature hypothesis. However, the study of the effects of IL-33 on the different aspects of the physiopathology of hematological malignancies could help us to better understand the intimate mechanisms that regulate the onset and progression of these diseases and make their treatment easier.

## Figures and Tables

**Table 1:** Pro/antitumorigenic role of IL-33 in hematological diseases.

Disease	Model Used	Pro/Anti
BCR-ABL1-negative myeloproliferative neoplasms neoplasms [68,69]	Cell lines Animal models	pro
Lymphomas [70,71,72,73]	Cell lines Mouse model Patients tissues	pro/anti
Acute myeloid leukemia [52,74,75,76]	Cell lines Animal models	anti
Chronic myeloid leukemia [68,77,78]	Cell lines Animal models	pro
MM bone disease [79,80,81]	Cell lines Patients tissues	anti
